# Prime editing of the common Familial Dysautonomia-causing c.2204 + 6T > C splicing mutation

**DOI:** 10.1186/s13023-026-04292-8

**Published:** 2026-04-10

**Authors:** Laura Peretto, Mirko Pinotti, Dario Balestra

**Affiliations:** Department of Life Sciences and Biotechnology, Laboratory for Advanced Therapies (LTTA), and Laboratory for Advanced Therapies (LTTA), Via Fossato di Mortara 74, Ferrara, 44121 Italy

**Keywords:** Familial Dysautonomia, *IKAP* c.2204 + 6t > c mutation, Prime editing

## Abstract

Familial Dysautonomia (FD, OMIM #223900) is a rare, life-threatening autosomal recessive neuropathy caused in 99.8% of patients by the c.2204 + 6T > C intronic mutation in the *ELP1/IKAP* gene. This substitution induces exon 20 skipping, leading to reduced ELP1 expression. While splicing-modulating therapies have shown partial efficacy, a permanent genetic correction remains unavailable. Here, we report the first application of Prime Editing (PE) to rescue the FD-causing *IKAP* splicing defect. Using a mutant exon-trapping minigene (pTB-*IKAP*) transiently co-transfected in HEK293T cells with PE2 or PE3 components, we demonstrate a significant increase in exon 20 inclusion, from 19 ± 2% to 48 ± 3% and 60 ± 3%, respectively. Restriction fragment length polymorphism and Sanger sequencing confirmed correction of the mutant allele, with PE3 achieving ~ 10% genomic editing efficiency. Moreover, by targeting ESS2 via a silent A > G substitution, we similarly restored exon inclusion to 50 ± 4%. These findings provide proof-of-principle that prime editing, particularly PE3, can efficiently correct or bypass the *ELP1/IKAP* c.2204 + 6T > C mutation and restore proper splicing. Given that modest increases in ELP1 expression (5–10% of wild-type) markedly alleviate FD severity in mouse models, our results highlight PE as a promising, potentially curative approach and lay the foundation for future ex-vivo and in vivo studies.

**Supplementary Information:**

The online version contains supplementary material available at 10.1186/s13023-026-04292-8.

Familial Dysautonomia (FD, OMIM #223900), also known as Riley-Day syndrome, is a rare autosomal recessive disorder characterized by progressive degeneration of the sensory and autonomic nervous system. The disease mainly affects the Ashkenazi Jew population, with a carrier frequency of 1 in 32 and 1 in 19 in Jews of Polish descent and occurs in approximately 1 in 3,600 live births in Eastern European Jewish extraction [1]. FD is considered a life-threatening condition with a high fatality rate, and there is no cure but only therapies to alleviate symptoms [2].

For almost all patients (99.8%) [3, 4] FD is caused by the c.2204 + 6T > C intronic mutation in the *ELP1/IKAP* gene that encodes the elongator complex protein 1 (ELP1), also known as the I kappa B kinase complex-associated protein (IKBKAP or IKAP). ELP1 is a well-conserved protein implicated in various processes, including cell migration, Jun N-terminal kinases (JNK) signalling, exocytosis, and tRNA modification [5].

The *ELP1/IKAP* c.2204 + 6T > C substitution, by disrupting the donor splice site of exon 20, induces exon 20 skipping and frameshift [6], and two exonic splicing silencers, ESS1 and ESS2 located in exon 20, repress *ELP1/IKAP* exon 20 inclusion through the interaction with hnRNP A1 [7]. Interestingly, by unclear mechanisms, the aberrant splicing patterns and the normal/mutant transcript ratio are tissue-specific, with the lowest values found in neuronal tissues with low ELP1/IKAP expression levels [8].

To restore proper ELP1/IKAP exon 20 inclusion different splicing-switching molecules have been developed, and their efficacy has been demonstrated in vitro, ex vivo, and in mouse models [9–11], and a clinical trial is ongoing [12]. However, these approaches have not yet optimized, and several open issues still remain.

In this context genome editing could represent a permanent cure with a single intervention. Among the several gene therapy approaches that can be potentially applied to neurogenetic disorders [13], the recently developed Prime Editing (PE), that is a ‘search-and-replace’ genome editing technology, represents an attracting strategy. As a matter of fact, PE can mediate targeted insertions, deletions, and all possible base-to-base conversions in human cells without requiring DSBs or donor DNA templates [14]. For the PE2 system the basic components are a fusion protein including a Cas9 nickase and an engineered reverse transcriptase (RT) domain, and a prime editing guide RNA (pegRNA) that not only indicate the target site but also encodes for the desired modification. An additional RNA guide targeting the opposite strand is used in the PE3 system (with PE editor, the pegRNA and the gRNA), to increase the editing efficiency. This strategy directs the cell’s endogenous mismatch repair (MMR) machinery to use the edited strand (containing the desired modification) as a template for repair, thereby significantly increasing the permanent incorporation of the edit compared to the PE2 system [14].

In the present study we explored for the first time PE to rescue the ELP1/IKAP exon 20 inclusion impaired by the FD-causing *IKAP* c.2204 + 6T > C mutation.

As experimental in vitro model we transiently transfected the well-established exon-trapping ELP1/IKAP mutant minigene (pTB-IKAP) in HEK293T cell line, a highly transfectable cellular model largely exploited during landmark studies in the field of PE development (Supp. Figure 1A) [10, 14, 15], and evaluated the rescue by PE at the DNA and mRNA level. Through different bioinformatic tools (CRISPR RGEN Tools; http://www.rgenome.net/http://www.rgenome.net/) we designed a pegRNA and a ngRNA (Supp. Figure 1B) to correct the causative mutation via PE2 and PE3 approaches [15] exploiting a prime editor that recognizes an NGG-PAM. To assess potential off-target activity, we performed a comprehensive computational prediction of candidate sites for both the pegRNA spacer and the nicking gRNA in the human genome, applying highly conservative filtering criteria informed by recent experimental evidence on prime editing specificity [14, 16, 17]. Notably, none of the predicted off-target loci displayed the requisite features to support functional prime editing: the vast majority exhibited at least one mismatch within the 12-nucleotide seed region proximal to the PAM (positions 8–20 of the spacer), a condition known to severely impair Cas9 binding and subsequent editing. In the few instances where the seed region was fully matched, the Primer Binding Site (PBS) consistently harbored three or more mismatches relative to the genomic target, a degree of divergence demonstrated to reduce prime editing efficiency by over 90% even with a single mismatch [18]. Based on these lines of evidence, we considered the off-target risk in our system to be negligible (supplementary Table 1).

These expression vectors were then created and co-transfected with the mutant minigene. Total RNA was extracted seventy-two hr after transfection to analyse splicing patterns through RT-PCR with pTB-specific primers. As expected, the splicing pattern of the mutated minigene alone confirmed the exon 20 skipping event with a remarkably reduced proportion of correctly spliced transcripts (19 ± 2%) (Fig. 1A). Noticeably, this proportion significantly increased upon co-transfection with the PE2 (48 ± 3%) and particularly with PE3 (60 ± 3%).

To provide direct experimental evidence of the correction of the best performing PE3 system at the DNA level on treated cells the region of interest was PCR amplified and digested with HaeII that specifically recognizes the mutated site (RFLP). As shown in Fig. 1B, the undigested band, whose wild-type features were demonstrated by Sanger sequencing, was appreciable in the mutated condition only upon PE3 treatment. This result was further confirmed by denaturing capillary electrophoresis of the fluorescently-labelled amplicons undergoing digestion, which led to properly estimate the correction around 10% in the absence of the heteroduplex DNA impairing restriction (Fig. 1C). The discrepancy between the correction at DNA and RNA levels is a common observation in splicing-correction studies utilizing minigenes and can be attributed to two main factors. First, the sensitivity limits of conventional detection methods—such as Sanger sequencing and restriction fragment length polymorphism (RFLP) analysis—typically impose a detection threshold of approximately 5%. Thus, PE2 editing efficiency might fall just below this threshold at the DNA level, yet still generate sufficient corrected mRNA to be detected by the highly sensitive RT-PCR. Second, an amplification effect arises from the fact that a single corrected DNA molecule can serve as a template for numerous rounds of transcription, leading to an over-representation of the corrected signal at the mRNA level. This phenomenon has been previously documented in other gene editing applications targeting splicing [19]. As an alternative strategy we exploited the PE approach to abolish the ESS2 by introducing a silent change A > G (Supp. Figure 1A). As shown in Fig. 1D, co-transfection of the mutant minigene with the PE2 or the PE3 components rescued the exon 20 inclusion to 30 ± 4% or 50 ± 4%, respectively. The presence of the targeted nucleotide in the mature mRNA led also us to confirm the desired change via Sanger sequencing and analysis of peaks, with an estimated correction efficiency of approximately 30% with PE3.

**Fig. 1:**
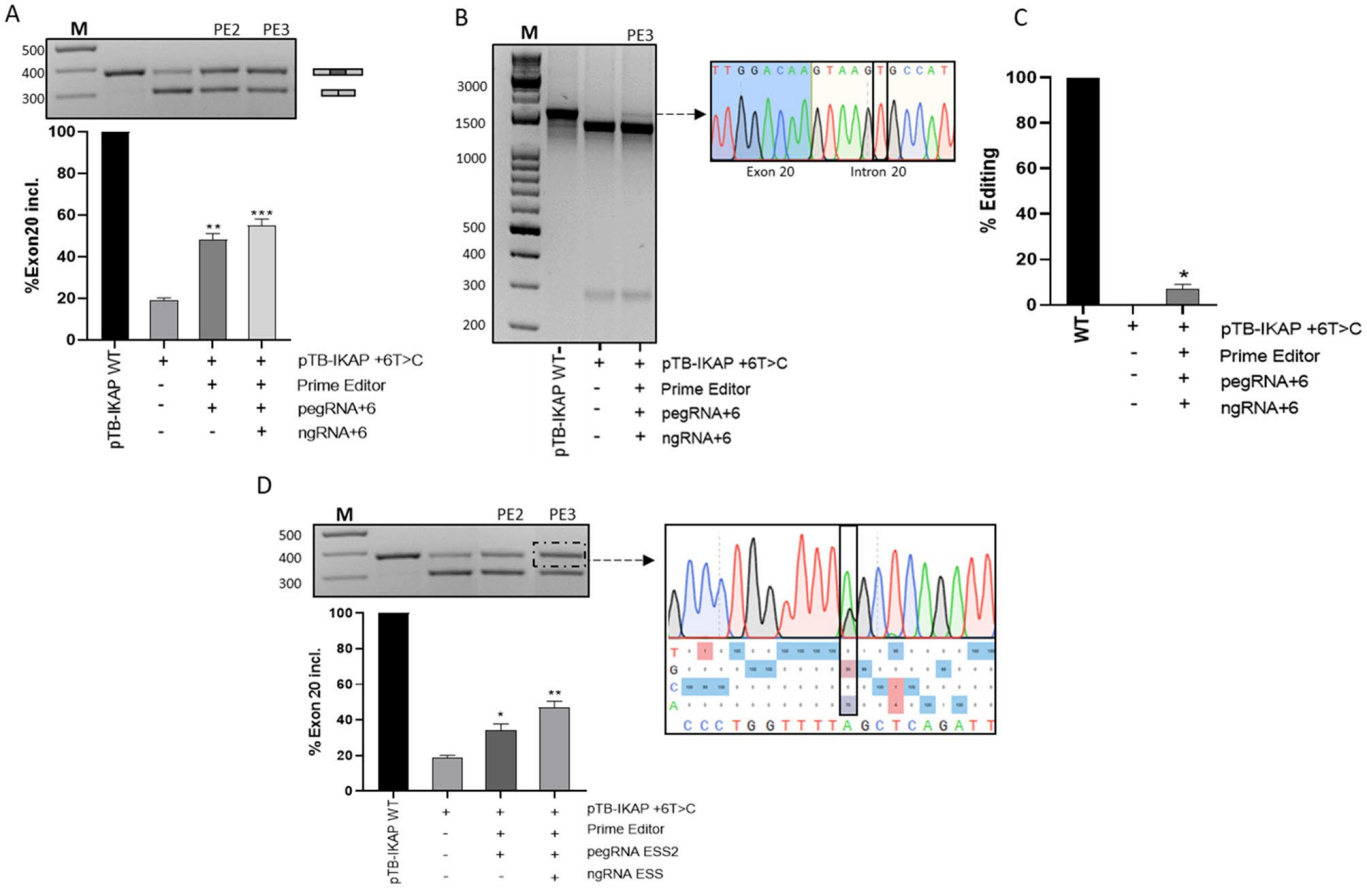
Correction of the c.2204 + 6T > C mutation or abolition of the ESS2 by prime editing. **A**) Targeting of the mutation by PE and evaluation of correctly spliced (349 bp) or exon 20-skipped (275 bp) transcripts by RT-PCR followed by 2% agarose gel electrophoresis (upper panel) and quantification of their relative proportion by imagej (lower panel). The schematic representation of the transcripts is reported on the right. M: 100 bp molecular marker. **B)** Restriction fragment length polymorphism (RFPL) analysis upon HaeII digestion followed by 2% agarose gel electrophoresis (left panel). The right panel reports the Sanger sequencing of the undigested amplicon from the edited samples. **C**) Histograms reports the relative proportion of the undigested fragment obtained through the denaturing capillary electrophoresis of fluorescently-labelled PCR products. **D**) Targeting of the ESSE2 by PE and evaluation of correctly spliced or exon 20-skipped transcripts by RT-PCR followed by 2% agarose gel electrophoresis (upper-left panel) and quantification of their relative proportion by imagej (lower-right panel). The right panel reports the Sanger sequencing of the correctly-spliced transcripts after PE3-mediated correction with the inserted change shown in the black rectangle. The percentages showed below indicate the quantifications of the picks obtained using EditR software. Results from three independent experiments (mean and standard deviation) are shown. The Student’s t-test with Welch’s corrections was used for statistical analysis, with *p* > 0.05 considered not significant (ns); *p* < 0.05*; *p* < 0.01**; *p* < 0.001***

In conclusion, this pioneer study, although conducted exclusively on minigene which could not fully reflect the genomic contexts, provides the first proof of principle that PE, and particularly the most efficient PE3, can be effectively exploited to counteract the FD-causing *ELP1/IKAP* c.2204 + 6T > C change and rescue exon 20 inclusion by either reverting the mutation or abolishing an exonic splicing silencer. It is worth noting that the extent of rescue at the DNA and mRNA splicing level could have a pathophysiologic relevance since a slight increase in ELP1/IKAP expression (5–10% of wt) in FD mouse models (transgenic ikbkapΔ20/flox) appears to significantly reduce FD severity and increase survival [8].

Altogether these preliminary data pave the way for further studies in ex vivo and particularly in vivo FD models to test delivery of the PE components to nervous systems via viral and non-viral methods, or a combination thereof [20], and assess the therapeutic potential, and safety profile (i.e. off-targets) of this promising strategy [17]. Data on FD would pave the way to apply PE for other rare, orphan, hereditary sensory and autonomic neuropathies.

## Electronic Supplementary Material

Below is the link to the electronic supplementary material.

Supplementary Material 1

Supplementary Material 2

## Data Availability

Data are contained within the article and Supplementary Materials.

## Abbreviations

A > G: Adenine-to-Guanine substitution
Cas9: CRISPR-associated protein 9
cDNA: Complementary deoxyribonucleic acid
CRISPR: Clustered regularly interspaced short palindromic repeats
DSB: Double-strand break
ELP1: Elongator complex protein 1
ESS: Exonic splicing silencer
FD: Familial Dysautonomia
HEK293T: Human embryonic kidney 293T cell line
hnRNP A1: Heterogeneous nuclear ribonucleoprotein A1
ELP1/IKAP: IκB kinase complex-associated protein (encoded by ELP1/IKAP gene)
JNK: c-Jun N-terminal kinase
mRNA: Messenger ribonucleic acid
ngRNA: Nicking guide RNA
OMIM: Online Mendelian Inheritance in Man
PAM: Protospacer adjacent motif
PCR: Polymerase chain reaction
PE: Prime Editing
PE2 / PE3: Prime Editing system 2 / 3
pegRNA: Prime editing guide RNA
RFLP: Restriction fragment length polymorphism
RT: Reverse transcriptase
T > C: Thymine to Cytosine substitution
tRNA: transfer RNA
wt: Wild type

## References

[1] Norcliffe-Kaufmann L, Slaugenhaupt SA, Kaufmann H. Familial dysautonomia: History, genotype, phenotype and translational research. Prog Neurobiol. 2017;152:131–48. 27317387 10.1016/j.pneurobio.2016.06.003

[2] Jackson MZ, Gruner KA, Qin C, Tourtellotte WG. A neuron autonomous role for the familial dysautonomia gene ELP1 in sympathetic and sensory target tissue innervation. Development. 2014;141(12):2452–61. 24917501 10.1242/dev.107797 PMC4050699

[3] Anderson SL, Coli R, Daly IW, Kichula EA, Rork MJ, Volpi SA, et al. Familial dysautonomia is caused by mutations of the IKAP gene. Am J Hum Genet. 2001;68(3):753–8. 11179021 10.1086/318808 PMC1274486

[4] Slaugenhaupt SA, Blumenfeld A, Gill SP, Leyne M, Mull J, Cuajungco MP, et al. Tissue-specific expression of a splicing mutation in the IKBKAP gene causes familial dysautonomia. Am J Hum Genet. 2001;68(3):598–605. 11179008 10.1086/318810 PMC1274473

[5] Blumenfeld A, Slaugenhaupt SA, Axelrod FB, Lucente DE, Maayan C, Liebert CB, et al. Localization of the gene for familial dysautonomia on chromosome 9 and definition of DNA markers for genetic diagnosis. Nat Genet. 1993;4(2):160–4. 8102296 10.1038/ng0693-160

[6] Boone N, Loriod B, Bergon A, Sbai O, Formisano-Tréziny C, Gabert J, et al. Olfactory stem cells, a new cellular model for studying molecular mechanisms underlying familial dysautonomia. PLoS ONE. 2010;5(12):e15590. 21187979 10.1371/journal.pone.0015590 PMC3004942

[7] Bruun GH, Bang JMV, Christensen LL, Brøner S, Petersen USS, Guerra B, et al. Blocking of an intronic splicing silencer completely rescues IKBKAP exon 20 splicing in familial dysautonomia patient cells. Nucleic Acids Res. 2018;46(15):7938–52. 29762696 10.1093/nar/gky395 PMC6125618

[8] Dietrich P, Alli S, Shanmugasundaram R, Dragatsis I. IKAP expression levels modulate disease severity in a mouse model of familial dysautonomia. Hum Mol Genet. 2012;21(23):5078–90. 22922231 10.1093/hmg/dds354 PMC3490515

[9] Romano G, Riccardi F, Bussani E, Vodret S, Licastro D, Ragone I, et al. Rescue of a familial dysautonomia mouse model by AAV9-Exon-specific U1 snRNA. Am J Hum Genet. 2022;109(8):1534–48. 35905737 10.1016/j.ajhg.2022.07.004 PMC9388384

[10] Donadon I, Pinotti M, Rajkowska K, Pianigiani G, Barbon E, Morini E, et al. Exon-specific U1 snRNAs improve ELP1 exon 20 definition and rescue ELP1 protein expression in a familial dysautonomia mouse model. Hum Mol Genet. 2018;27(14):2466–76. 29701768 10.1093/hmg/ddy151 PMC6030917

[11] Sinha R, Kim YJ, Nomakuchi T, Sahashi K, Hua Y, Rigo F, et al. Antisense oligonucleotides correct the familial dysautonomia splicing defect in IKBKAP transgenic mice. Nucleic Acids Res. 2018;46(10):4833–44. 29672717 10.1093/nar/gky249 PMC6007753

[12] Grobocopatel Marra M, Kuijpers M, Kaufmann H, Gonzalez-Duarte A. Advances in the treatment of familial dysautonomia: what does the future hold? Expert Rev Neurother. 2025;25(8):939–49. 40580154 10.1080/14737175.2025.2525400

[13] Devinsky O, Coller J, Ahrens-Nicklas R, Liu XS, Ahituv N, Davidson BL, et al. Gene therapies for neurogenetic disorders. Trends Mol Med. 2025;31(9):814–26. 39966070 10.1016/j.molmed.2025.01.015

[14] Anzalone AV, Randolph PB, Davis JR, Sousa AA, Koblan LW, Levy JM, et al. Search-and-replace genome editing without double-strand breaks or donor DNA. Nature. 2019;576(7785):149–57. 31634902 10.1038/s41586-019-1711-4 PMC6907074

[15] Chen PJ, Hussmann JA, Yan J, Knipping F, Ravisankar P, Chen PF, et al. Enhanced prime editing systems by manipulating cellular determinants of editing outcomes. Cell. 2021;184(22):5635–e565229. 34653350 10.1016/j.cell.2021.09.018 PMC8584034

[16] Mathis N, Allam A, Kissling L, Marquart KF, Schmidheini L, Solari C, et al. Predicting prime editing efficiency and product purity by deep learning. Nat Biotechnol. 2023;41(8):1151–9. 36646933 10.1038/s41587-022-01613-7 PMC7614945

[17] Lee J, Kweon J, Kim Y. Emerging trends in prime editing for precision genome editing. Exp Mol Med. 2025;57(7):1381–91. 40744998 10.1038/s12276-025-01463-8 PMC12322275

[18] Nelson JW, Randolph PB, Shen SP, Everette KA, Chen PJ, Anzalone AV, et al. Engineered pegRNAs improve prime editing efficiency. Nat Biotechnol. 2022;40(3):402–10. 10.1038/s41587-021-01039-7 PMC8930418 34608327

[19] Schiroli G, Ferrari S, Conway A, Jacob A, Capo V, Albano L, et al. Preclinical modeling highlights the therapeutic potential of hematopoietic stem cell gene editing for correction of SCID-X1. Sci Transl Med. 2017;9(411):eaan0820. 29021165 10.1126/scitranslmed.aan0820

[20] Salomonsson SE, Clelland CD. Building CRISPR Gene Therapies for the Central Nervous System: A Review. JAMA Neurol. 2024;81(3):283–90. 38285472 10.1001/jamaneurol.2023.4983 PMC11164426

