# Supplementary material for: Prime editing of the common Familial Dysautonomia-causing c.2204 + 6T > C splicing mutation
Source: Orphanet J Rare Dis. 2026 Apr 10;21:144. doi: 10.1186/s13023-026-04292-8 (PMC13067553; doi:10.1186/s13023-026-04292-8)
Supplement: Supplementary file 1 — Supplementary Material 1 [file 13023_2026_4292_MOESM1_ESM.docx]

Potential off-target sites for the pegRNA and ngRNA spacers were identified using CRISPOR (Concordet & Haeussler, 2018; http://crispor.tefor.net). Each 20‑nt spacer sequence was queried against the reference genome [Homo sapiens, hg19] allowing up to 4 mismatches in the protospacer and considering the canonical NGG PAM.

For each predicted site, the following information was recorded:

-genomic location (chromosome, coordinates, strand),

-full protospacer + PAM sequence,

-total number and positions of mismatches relative to the spacer,

-total number and positions of mismatches relative to the PBS,

Two successive filtering steps were applied, based on specificity determinants of prime editing:

Filter 1 – Integrity of the extended seed region (12 nt)

All sites harbouring any mismatch within positions 8–20 of the protospacer (the 12 nucleotides immediately upstream of the PAM) were discarded. This criterion stems from the observation that stable Cas9 binding and subsequent reverse transcription require near‑perfect pairing in this region (Mathis et al., 2020; Anzalone et al., 2019; Lee et al., 2025).

Filter 2 – Complementarity of the Primer Binding Site (PBS)

For sites that passed Filter 1 (i.e., no mismatches in the 12‑nt seed), we extracted the 20‑nt genomic sequence immediately upstream of the PAM and calculated the number of mismatches with the reverse complement of the pegRNA PBS sequence. A site was considered potentially hazardous only if it displayed ≤1 mismatch in the PBS, in agreement with Nelson et al. (2022), who reported >95% loss of editing activity with as few as two PBS mismatches.

References:

- Concordet, J.P., Haeussler, M. (2018). CRISPOR: intuitive guide selection for CRISPR/Cas9 genome editing experiments and screens. Nucleic Acids Research, 46(W1), W242–W245.
- Anzalone, A.V. et al. (2019). Search-and-replace genome editing without double-strand breaks or donor DNA. Nature, 576, 149–157.
- Mathis N, Allam A, Kissling L, et al. Predicting prime editing efficiency and product purity by deep learning. Nat Biotechnol. 2023;41(8):1151-1159. doi:10.1038/s41587-022-01613-7
- Nelson, J.W. et al. (2022). Engineered pegRNAs improve prime editing efficiency. Nature Biotechnology, 40, 402–410.
- Lee J, Kweon J, Kim Y. Emerging trends in prime editing for precision genome editing. Exp Mol Med. 2025;57(7):1381-1391. doi:10.1038/s12276-025-01463-8.
- Cingolani, P. et al. (2012). A program for annotating and predicting the effects of single nucleotide polymorphisms, SnpEff. Fly, 6(2), 80–92.

| guideId | guideSeq | offtargetSeq | mismatchPos | seed 12 nucleotidi | possibile? | mismatchCount | mitOfftargetScore | cfdOfftargetScore | chrom | start | end | strand | locusDesc |
| --- | --- | --- | --- | --- | --- | --- | --- | --- | --- | --- | --- | --- | --- |
| 155rev | ACTAGTCGCAAACAGTACAATGG | ACTAAATACAAACAGTACAAAGG | ....****............ | ............ | Yes | 4 | 0,539223 | 0,610278 | chr7 | 1,2E+07 | 11511848 | - | intron:THSD7A |
| 155rev | ACTAGTCGCAAACAGTACAATGG | ATGAATCACAAACAGTACAATGG | .**.*..*............ | ............ | Yes | 4 | 1,345833 | 0,393939 | chr1 | 7E+07 | 70094549 | + | intergenic:RP11-379C1.1-LRRC7 |
| 155rev | ACTAGTCGCAAACAGTACAATGG | TCTGTTCTCAAACAGTACAAGAG | *..**..*............ | ............ | Yes | 4 | 0,277237 | 0,030382 | chr6 | 3938856 | 3938878 | - | intergenic:RP1-140K8.3-TDGF1P4 |
| 155rev | ACTAGTCGCAAACAGTACAATGG | TCCAGTCACAAACAGTACAAGGG | *.*....*............ | ............ | Yes | 3 | 2,569822 | 0,428571 | chr17 | 2,8E+07 | 28278798 | + | intron:EFCAB5 |
| 155rev | ACTAGTCGCAAACAGTACAATGG | AGTAGAGGCAAACAGTACAATGA | .*...**............. | ............ | Yes | 3 | 0,205257 | 0,019703 | chr7 | 3E+07 | 30348533 | + | intergenic:MIR550A1-ZNRF2 |
| 155rev | ACTAGTCGCAAACAGTACAATGG | ACTCTTCCCAAACAGTACAAGAG | ...**..*............ | ............ | Yes | 3 | 0,485313 | 0,016893 | chr12 | 4,4E+07 | 43760973 | + | intron:ADAMTS20 |
