# Supplementary material for: Prime editing of the common Familial Dysautonomia-causing c.2204 + 6T > C splicing mutation
Source: Orphanet J Rare Dis. 2026 Apr 10;21:144. doi: 10.1186/s13023-026-04292-8 (PMC13067553; doi:10.1186/s13023-026-04292-8)
Supplement: Supplementary file 2 — Supplementary Material 2 [file 13023_2026_4292_MOESM2_ESM.docx]

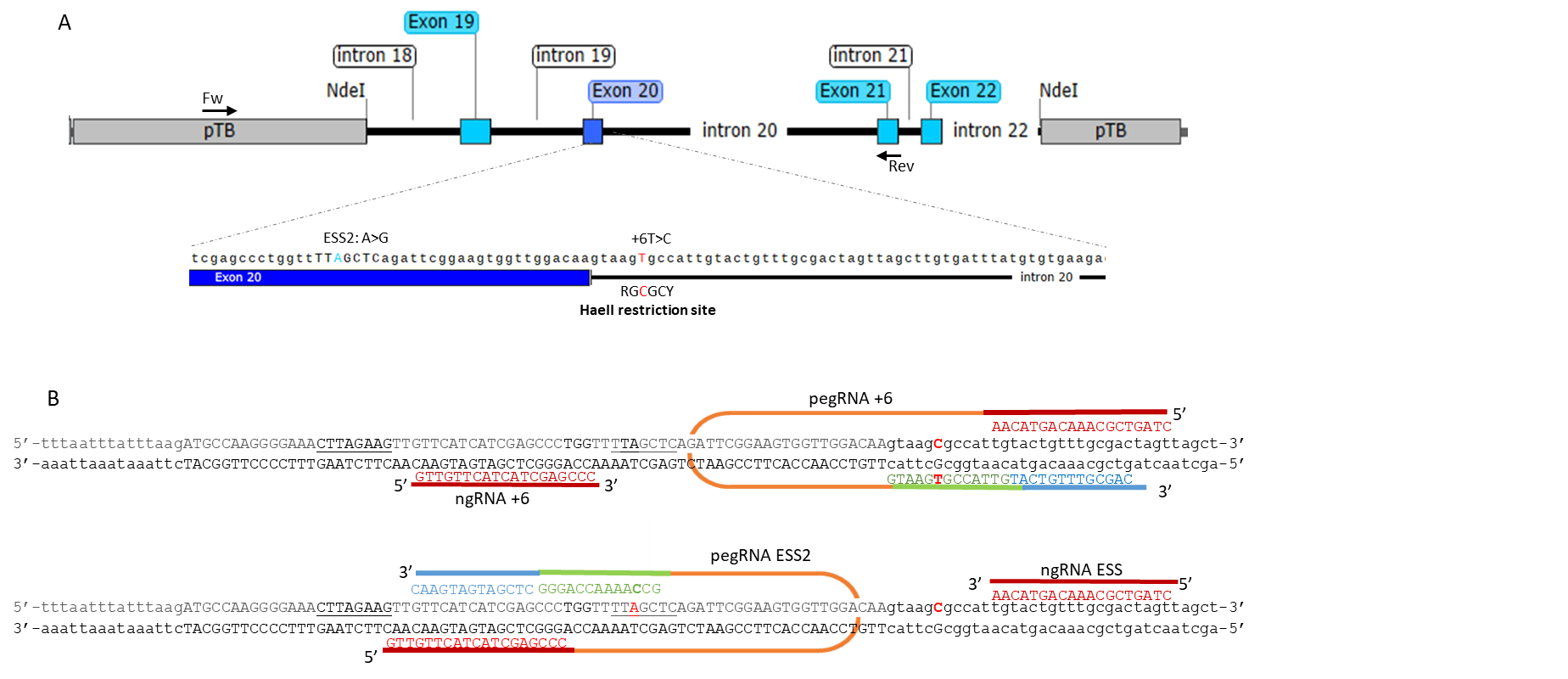


***LEGEND TO THE SUPPLEMENTARY FIGURE 1***

***A)*** *Schematic representation of the pTB-IKAP minigene (upper panel). Exonic and intronic sequences are represented by boxes and lines, respectively. The Arrows represent primers used for RT-PCR amplifications. The lower panel reports the region of interest where the targeted nucleotides are in light blue and in red. The consensus sequence recognized by the restriction enzyme used for the RFLP is reported below.*

***B)*** *Schematic representation of the designed pegRNAs and ngRNAs. The sequence of the sense and antisense DNA strands, with exonic and intronic sequences in upper and lower case, respectively, is reported. The targeted nucleotides are in red. The spacer RNA, and the PBS, RTT, and sgRNA scaffold sequences are indicated in red, blue, green, and orange, respectively.*
